# Radiation-induced skin injury: pathogenesis, treatment, and management

**DOI:** 10.18632/aging.103932

**Published:** 2020-11-16

**Authors:** Xiaojing Yang, Hanru Ren, Xiaomao Guo, Chaosu Hu, Jie Fu

**Affiliations:** 1 Department of Radiation Oncology, Shanghai Jiao Tong University Affiliated Sixth People’s Hospital, Shanghai, China; 2 Department of Orthopedics, Shanghai Pudong Hospital, Fudan University, Pudong Medical Center, Shanghai, China; 3 Department of Radiation Oncology, Fudan University Shanghai Cancer Center, Shanghai, China; 4 Shanghai Medical College, Fudan University, Shanghai, China; 5 Department of Oncology, Shanghai Medical College, Fudan University, Shanghai, China

**Keywords:** radiation, skin injury

## Abstract

Radiation-induced skin injury (RSI) refers to a frequently occurring complication of radiation therapy. Nearly 90% of patients having received radiation therapy underwent moderate-to-severe skin reactions, severely reducing patients' quality of life and adversely affecting their disease treatment. No gold standard has been formulated for RSIs. In the present study, the mechanism of RSI and topical medications was discussed. Besides, this study can be referenced for clinicians to treat RSIs to guide subsequent clinical medicine.

## INTRODUCTION

Radiation therapy can be adopted to effectively malignant tumors. Radiation not only has a killing effect on tumor cells but also has a powerful destructive effect on normal tissue cells in the irradiation field. During radiotherapy treatment, to a certain extent, a wide range of radiation doses, various radiation, energy of the radiation, treatment time of the radiation, and course of treatment overall affected the patient. Patients having undergone radiotherapy may develop different skin damage as impacted by their different ages, physical conditions, skin types, as well as location and duration of exposure. Numerous advanced radiotherapy technologies for tumors have developed rapidly and been progressively applied in clinics. Despite the increasing accuracy of radiation therapy, normal tissues are still unavoidably exposed. The main causes of RSI include nuclear radiation accidents, tumor radiotherapy, and occupational exposure. In tumor radiotherapy, the incidence of RSIs has also been gradually elevated, and nearly 85%–95% of tumor patients have developed different degrees of skin damage attributed to radiotherapy. Accordingly, the quality of life is seriously deteriorated, and huge psychological and economic pressure is exerted on the patient, while radiotherapy, and thus, the treatment are interrupted [1, 2]. On the whole, RSIs consist of two types, i.e., acute and chronic. Acute RSIs involve dry and wet desquamation, skin necrosis, ulcers, as well as bleeding [3]. Chronic RSIs cover chronic ulcers, radiation-induced keratosis, telangiectasias, fibrosis, as well as skin cancer [4]. Compared with skin damage that is attributed to other factors, RSI is characterized by incubation period, timeliness, potentiality, progress and persistence. Unlike ordinary burns and ulcers, radiation directly damages the skin as well as its deep tissue cells, causing dryness, loss of elasticity, pigmentation, soft tissue fibrosis, capillary dilatation, and radiation dermatitis in irradiated areas. Moreover, it irreversibly damages microvascular and small blood vessel endothelial cells in skin tissue. As a result, patients’ damaged skin does not heal for a long time, and it exhibits susceptibility to infection. The lesion eventually develops into fibrosis of the skin tissue and even becomes cancerous, significantly deteriorating patients’ quality of life. Numerous existing medicaments and dressings worldwide are available to prevent and treat radioactive skin damage (e.g., corticosteroids, hyaluronic acid, triethanolamine, sucralfate cream, aloe, calendula cream, as well as water-based cream). The conclusions of various studies often appear to contradict each other and lack universality for the lack of high-quality large-sample studies and uniform assessment standards. Clinically, the prevention and management of RSI is commonly based on personal experience, without scientific evidence [5].

## Pathophysiology and mechanism

### Fibrosis

The pathophysiological variations in RSI consist of erythema and desquamation that occur shortly, as well as chronic skin atrophy, ulcers, telangiectasias and fibrosis [6, 7]. Progressive endometritis occurs for gradual occlusion of the microvasculature and hypoxia attributed to fibrosis. The wound healing in the illuminated area is limited as impacted by the disruption of the natural process of overlapping wound healing [8]. Radiation fibrosis refers to a harmful chronic disease that appears weeks to years after radiation [8]. Despite the rapid development of radiation therapy technology, radiation fibrosis is an irreversible procedure that significantly impairs the progress of radiation therapy and reduces the quality of life of patients [9]. Pandya et al. [10] tested specimens from 27 patients with oral squamous cell carcinoma who underwent radiation therapy for the jaw and neck. They reported significant tissue atrophy and atypical hyperplasia, increased fibrous exudative necrosis, thickened blood vessel walls, as well as dilated oropharynx of the salivary glands. Moreover, for the same patients, dense fibrosis with thick fibers was commonly identified in post-radiation tissues. It was inferred that similar findings elsewhere were due to increased tissue fibrosis and hypoxia for microvascular damage. Glands in the dermis were similarly damaged. The mentioned findings again confirmed the characteristics of microvascular thrombosis and tissue fibrosis of the final ulcer attributed to skin variations after radiation [11]. Radiation fibrosis is a sophisticated reaction that involves multiple stages. It consists of inflammation, proliferation and remodeling. It is an abnormal wound healing process attributed to the imbalance of proinflammatory and profibrotic cytokines [12]. Increased connective tissue causes fibrosis and leads to organ dysfunction [13]. Fibroblast-derived myofibroblasts critically impact fibrosis development by continuously synthesizing ECM and secreting type I collagen and α-smooth muscle actin [14]. Facilitated synthesis and deposition of ECM and accumulation of fibroblasts are considered the characteristics of skin fibrosis. Several mechanisms are involved in skin fibrosis, including fibroblast differentiation [15], epithelial-to-mesenchymal transition (EMT), [16] and leukocyte recruitment [11].

**Figure 1:**
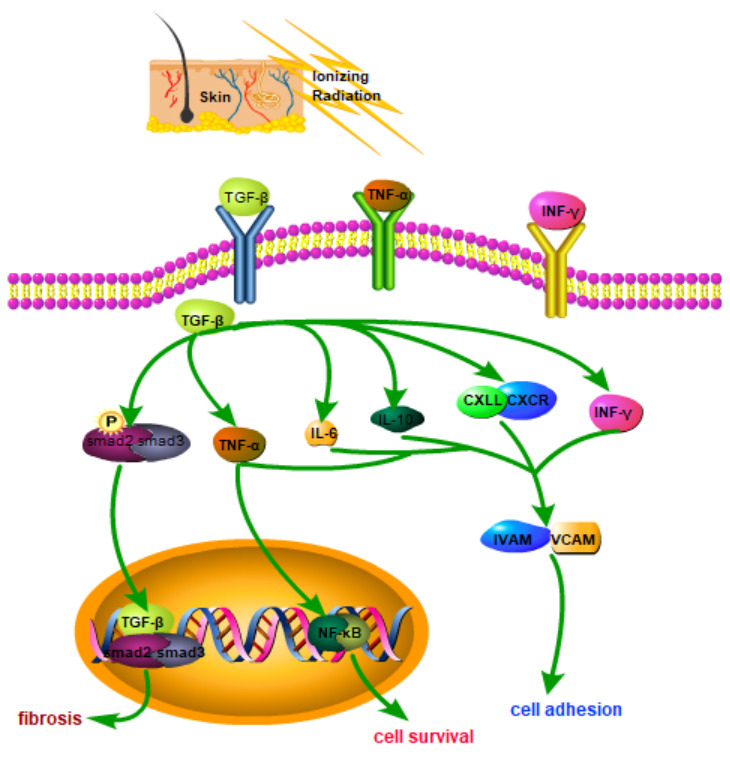
**Schematic diagram of related molecular mechanisms that may be involved in RSI.**

Tissue damage repair and subsequent fibrosis involve multiple molecules and signaling pathways (e.g., transforming growth factor (TGF)-β, and Wnt/β-catenin) [17, 18]. TGF-β acts as a clear fibrosis driver. Radiation-induced TGF-β is expressed in skin tissue in a radiation dose–dependent manner [19]. TGF-β is combined with its receptor to form a trimeric complex, causing tissue fibrosis [20]. The TGF-β/Smad pathway is a significant signaling pathway involved in skin fibrosis. Activated Smad protein leads to the nucleus translocation, activates specific transcription, and triggers fibrosis in the nucleus [21]. Activated TGF-β regulates fibrotic target genes by phosphorylating Smad2/Smad3 proteins. The TGF-β signaling pathway has acted as a therapeutic target for radiation fibrosis [22]. Ionizing radiation is exerted on skin cells to cause apoptosis and generate free radicals and reactive oxygen species, primarily causing skin damage. The Wnt/β-catenin signaling pathway is vital to the physiological processes of early embryonic development, organ formation, and tissue regeneration in animals. Mutations in vital proteins in this signaling pathway can cause abnormal signal transduction, causing abnormal development or tissue regeneration [23]. A schematic is presented in Figure 1.

### Changes of skin lipid metabolism

Skin fat represents the main building block of human skin. Skin lipids have a radioprotective role. Radiation modulates skin lipid metabolism by downregulating multiple pathways. It also reduces the amount of skin fat and variations in lipid metabolism. Mature adipocytes promote the migration of co-cultured keratinocytes and fibroblasts, but do not promote their proliferation. Fatty acid–binding protein 4 can be incorporated into skin cells and promote the repair of DNA damage in irradiated skin fibroblasts. Radiation induces skin lipid remodeling, and skin fat cells have protective effects on radiation-induced skin damage [24]. Radiation-induced skin damage is depicted by a chronic inflammatory state and an increase in ROS synthesis. Ionizing radiation facilitates the synthesis of reactive nitrogen and oxygen species (RNS/ROS) for the radiolysis of water [25]. The mentioned reaction can induce oxidative damage and cytotoxicity, thereby causing acute or chronic skin damage. The use of antioxidants can reduce the damage attributed to radiation [26]. For instance, superoxide dismutase and its mimetics reduce ROS levels and RSI. Rare is known regarding the underlying mechanisms by which radiation generates and amplifies ROS. Nitric oxide (NO) is critical to the homeostasis of the functioning of the skin and has become the target of treatment for specific skin diseases [27]. In mammals, NO is synthesized by L-arginine, NADPH, as well as NO synthase (NOS) in oxygen. NOS isoforms are identified in the skin, and 5,6,7,8-tetrahydrobiopterin (BH4) acts as an important cofactor for NOS [28]. ROS synthesis may hinder the use of BH4 for the oxidation of BH4 to dihydrobiopterin (BH2). Accordingly, uncoupling of NOS may be caused, and the synthesis of oxidative superoxide radicals can be facilitated [29]. The inhibition of GCH1 *in vivo* increases oxidative stress and down-regulates the white blood cell count after radiation [30]. Radiation destroys BH4, thereby enhancing the ROS cascade response. GCH1 revives BH4 levels and ROS synthesis [31].

### Apoptosis

Ionizing radiation is capable of affecting G2- and M-phase cells in the cell cycle, thereby causing apoptosis and impaired cell proliferation and migration; as a result, an overall cell depletion is caused. Ionizing radiation can damage collagen structures. Cell proliferation is suppressed in irradiated wounds. Increased matrix metalloproteinases (MMPs) that are not counteracted by tissue inhibitors of MMPs (TIMP) cause abnormal degradation of ECM. Under decreased angiogenesis and increased transforming growth factor-β (TGF-β) levels, blood vessels show variations, causing increased endothelial fibrosis; subsequent occlusion of the vascular lumen causes tissue hypoxia. The low expression level of apoptosis-inhibiting gene *Ras* and the over-expression of apoptosis-inducing genes *p35* and others attributed to radiation during radiotherapy causes excessive apoptosis in patients’ bodies; thus, their skin is damaged. Existing studies reported that considerable radiation is generated during radiotherapy, and the reactive oxygen species and free radicals generated by the radiation can seriously damage the basal cells of the human body. Moreover, radiation inhibits the basal cell division and migration of keratinization function, inducing RSI in patients having received radiotherapy [32]. Moreover, radiation can lead to high expression levels of p53 and Bax proteins; it causes apoptosis and necrosis of local tissues (e.g., vascular endothelial cells, fibroblasts and epidermal cells); it adversely affects the process of neovascularization, wound margin contraction, as well as wound epidermalization [33]. Existing studies revealed that cytokines are directly or indirectly involved in radiation-induced damage [34]. IL-10 is capable of inhibiting the inflammatory response and reducing the activity of macrophages [34]. Neutrophils refer to the first cells that intrude the wound site within minutes after injury. They undergo apoptosis and are phagocytosed by macrophages 24–48 h after injury. The mentioned macrophages engulf cell debris and secrete growth factors that are critical to wound healing.

Ionizing radiation boosts the synthesis of reactive nitrogen and oxygen species (RNS/ROS) as impacted by the radiolysis of water [25]. The mentioned reaction causes oxidative damage and cytotoxicity, thereby causing acute or chronic skin damage. The use of antioxidants can mitigate the damage attributed to radiation [26]. For instance, superoxide dismutase and its mimetics down regulated ROS levels and RSI. The underlying mechanisms by which radiation generates and amplifies ROS are rarely known. Nitric oxide (NO) is critical to the homeostasis of the functioning of the skin, which has become the target of treatment for specific skin diseases [27]. In mammals, NO is synthesized by L-arginine, NADPH and NO synthase (NOS) in oxygen. NOS isoforms are identified in the skin, and 5,6,7,8-tetrahydrobiopterin (BH4) acts as a crucial cofactor for NOS [28]. The ROS synthesis may reduce the use of BH4 due to the oxidation of BH4 to dihydrobiopterin (BH2). This may cause uncoupling of NOS and lead to increased synthesis of oxidative superoxide radicals [29]. The inhibition of GCH1 *in vivo* increases oxidative stress and reduces the white blood cell count after radiation [30]. Radiation destroys BH4, thereby increasing the ROS cascade response. GCH1 revives BH4 levels and ROS synthesis [31].

### Changes of the process of neovascularization

Radiation can also cause a reduction in the expression levels of angiogenic factors. It up-regulates the expression levels of proinflammatory cytokines IL-1, IFN-γ, TNF-α, and IL-6, prevents collagen deposition, and induces TGF-beta1 expression by macrophage/stromal cell activation. Elevated levels of TGF-beta1 break down collagen and stimulate microvascular variations [8]. Neovascularization requires signaling through the vascular endothelial growth factor (VEGF) family [35]. VEGF refers to a marker of neovascularization. After exposure to 10 Gy irradiation, the synthesis of angiogenic factor VEGF in the blood of rat tumor carriers was significantly hindered [36]. Low levels of VEGF after radiotherapy indicated that targeted VEGF treatment enhanced vascular repair. Preclinical studies supported this by showing that irradiated rat bladder administrated with VEGF resulted in a marked reduction in fibrotic tissue and enhanced tissue angiogenesis [37]. PlGF refers to a member of the VEGF family and is involved primarily in pathological angiogenesis, including cancer. PlGF helps in wound healing by provoking blood vessel formation, macrophage recruitment, keratinocyte migration, and formation of granular tissue [38]. bFGF is an angiogenic growth factor with the ability to induce endothelial cell proliferation and migration. It is capable of expediting the healing of second-degree burn wounds and improving scar quality [39]. In burns, dermal components are required for surface resurfacing, and bFGF enhances wound healing and elevates the number of skin-derived mesenchymal stem cells in a dose-dependent manner under serum-free conditions [40]. In surgery, bFGF is immediately used for skin grafts and artificial dermal reconstruction after debridement [41]. Early use in local tissue may effectively protect radiation-damaged cells from cell death [42].

## General management

RSI management should start with patient education in skin care before, after and during radiation treatment (e.g., skin care, psychological care and diet care).

### Skin care

Skin care refers to the cleaning and care of patients’ skin, capable of effectively preventing wound infections as well as reducing the physical symptoms of discomfort to ensure the subsequent treatment of patients. Patient education should promote personal and wound hygiene, facilitate comfort, prevent trauma to the damaged skin, and manage radiation dermatitis. First of all, soft cotton clothing should be selected for the patient to prevent large friction to patients’ skin. Applying all kinds of irritating drugs or cosmetics is strictly prohibited. The body should not be scrubbed with soap, iodine, etc. Also, the use of ice or heat should prohibited. The hair in the exposed area of the body should not be shaved. The skin in the skin-irradiated area should be kept dry and clean. Certain basic hygiene habits are beneficial for managing radiation-induced skin toxicity. Intensive studies have been conducted on warm soapy water and warm water washing, which has now been recommended by clinicians [42]. Reports showed a marked reduction in itching and a lower RTOG radiation dermatitis score in moderate soap and water washing compared with no washing [43].

### Psychological care

Overall, patients have different levels of fear before receiving radiotherapy, which is quite normal. The nursing staff of the hospital should provide the patient knowledge regarding radiotherapy, various precautions, possible adverse reactions of various types in the body, and also skin care timely. Timely and effective communication can alleviate patients’ internal stress, and the patients can have a more relaxed attitude towards treatment.

### Diet care

A high-fat diet increases skin fat and increases resistance to RSI [24]. It is recommended that patients increase the amount of high-fat foods during treatment.

## Therapy

Methods and possible mechanisms for treating RSI are showed in Table 1.

**Table 1:** Methods and possible mechanisms for treating RSI.

**Treatment**	**References**	**Mechanism**
HBOT	Bassetto, F., et al. 2019 [44]	increase the oxygen supply, reduce the inflammatory exudation
Calendula	Gilca, M., et al. 2018 [45]	antibacterial, anti-inflammatory, antioxidant, and promote angiogenesis
Catechin	Scalia, S., et al. 2013 [47]	antioxidant
Aloe Vera	Surjushe, A., et al. 2008 [51]	anti-inflammatory
Chamomile	Aggag, M.E., et al. 1972 [53]	anti-inflammatory, antibacterial and antispasmodic
β-Sitosterol	Atiyeh, B.S., et al., 2002 [55]	analgesic, antibacterial, and anti-inflammatory
ASC	Aubertin, A. 1991 [57]	antioxidant
Pantothenic Acid	Aubertin, A. 1991 [57]	promote epithelial regeneration
HA	Liguori, V., et al. 1997 [59]	prevent ROS injury
EGF	Haubner, F., et al. 2012 [60]	induce the proliferation of fibroblasts, epidermal stem cells, and keratinocytes
GM-CSF	Cioffi, W.G., et al. 1991 [64]	lymphokine
PTX	Kumar, D., et al. 2018 [66]	anti-inflammatory; inhibit the TGF-β expression
Plasma	Lee, J., et al. 2019 [69]	enhance cell function through AKT signaling
Interleukins	Wei, J., et al. 2019 [70]	inflammatory
SODs	Kumar Soni, S., et al. 2019 [74]	endogenous enzymatic antioxidants
Triethanolamine cream	Lessmann, H., et al. 2009 [77]	reduce dryness, inflammation and edema
Corticosteroids	Haruna, F., et al. 2017 [78]	anti-inflammatory
Statins	Khattri, S., et al. 2013 [80]	immunomodulatory, anti-inflammatory, metabolic, antioxidant and antibacterial
Trolamine	Coulomb, B., et al. 1997 [83]	recruit macrophages and stimulate granulation tissue
Sucralfate	Kouloulias, V., et al. 2013 [88]	anti-inflammatory and antibacterial
SSD	Shanmugasundaram, N., et al. 2009 [89]	anti-inflammatory
Silver Nylon Dressing	Niazi, T.M., et al. 2012 [91]; Aquino-Parsons, C., et al. 2010 [92]	anti-inflammatory; barrier-enhancing
Silver-containing foam dressings with Safetac	Davies, P., et al. 2017 [93]	provide a moist healing environment

Abbreviations: RSI: Radiation-induced skin injury; HBOT: Hyperbaric oxygen therapy; ASC: Ascorbic Acid; PTX: Pentoxifylline; HA: Hyaluronic Acid; EGF: Epidermal Growth Factor; GM-CSF: Granulocyte Macrophage-Colony Stimulating Factor; SODs: Superoxide dismutases; SSD: Silver Sulfadiazine

### Physical therapy

### Hyperbaric oxygen therapy (HBOT)

HBOT refers to treatment with 100% oxygen at pressures above atmospheric pressure [44]. Studies have shown that the slow wound healing of patients results from hypoxemia for the fractured surface of the wound blood vessels. Oxygen therapy on skin lesions of patients can effectively increase the oxygen supply function of the skin lesions, reduce the inflammatory exudation of the wound, and accelerate the drying and healing of the wounds.

### Herbal

### Calendula

Calendula exerts antibacterial, anti-inflammatory and antioxidant effects, and it is capable of promoting angiogenesis. For this reason, it can repair wounds [45]. Pommier et al. delved into the effect of calendula on radiation-induced skin damage. Compared with triethanolamine, calendula significantly lowered the incidence of dermatitis. Moreover, patients administrated with calendula had fewer interruptions during radiation therapy and less radiation-induced pain [46].

### Catechins

Catechin is a natural phenolic compound. For its antioxidant activity, it can heal the skin damage from UV rays [47]. Epigallocatechin-3-gallate (EGCG) is the primary component of catechins. Studies have demonstrated its ability to inhibit radiation-induced damage to human skin cells and mouse skin [48]. EGCG protects cells from ROS by scavenging hydroxyl free radicals, superoxide anions, as well as hydrogen peroxide [49]. It can effectively mitigate radiation-induced damage. Clinical trials have confirmed the security of EGCG as well as its capability to avoid serious RSI. EGCG can continuously weaken tenderness, itching, pain and burns [50].

### Aloe vera

Aloe vera is considered a natural anti-inflammatory herb that mitigates radiation-induced skin damage [51]. Traditional Chinese medicine believes that RSI is primarily attributed to heat poisoning. Hence, heat treatment and detoxification should be employed as the major methods in traditional Chinese medicine treatment. As revealed from the results of traditional Chinese medicine treatment, evenly applying fresh aloe vera juice to the affected area daily can effectively mitigate the skin damage of patients. Aloe vera is not only cheap and effective, but also easy to use. Despite the mentioned encouraging properties, aloe vera has not been shown to decrease serious radiation-induced skin damage [52]. Compared with aqueous lotions in large randomized controlled trials (RCTs), it is less effective in alleviating patients' symptoms [52].

### Chamomile

Chamomile is derived from a medicinal plant with anti-inflammatory, antibacterial, and antispasmodic effects [53]. Despite the mentioned encouraging properties, the study of Ferreira et al. failed to illustrate the benefits of chamomile in treating RSI [54].

### β-Sitosterol

β-Sitosterol acts as a vital composition of sesame oil and beeswax. It is a herbal preparation with analgesic, antibacterial and anti-inflammatory components [55]. Compared with triethanolamine, it exhibits no major discrepancy to treat grade 2 and grade 3 dermatitis. However, the use of β-sitosterol significantly down regulated the incidence of severe itching and local skin pain [56].

### Topical vitamins

### Ascorbic Acid (ASC)

ASC (vitamin C) serves as an antioxidant and scavenges free radicals. ASC is capable of maintaining the enzymatic activity in patients' body, enhancing the tissue function of the biofilm and mitochondria, removing free radicals from the human body, and effectively treating the radiation-caused skin damage. Moreover, ASC can be involved in the normal metabolism of the human body, helping the body repair skin epithelial cells [57]. Halperin et al. delved into the workable shielding part of ASC in radiation therapy. However, the data failed to indicate any advantage of ASC in treating skin damage attributed to radiation therapy [57].

### Pantothenic Acid

Pantothenic acid (i.e., vitamin B5) is critical to metabolism and to maintain skin integrity. The lack of pantothenic acid can cause dermatitis, while its excess can promote epithelial regeneration. The data showed that topical fentanyl cream did not exert a protective effect on radiation-induced dermatitis [57].

### Endogenous agents

### Hyaluronic Acid (HA)

HA is a carbohydrate polymer throughout the connective tissue. It is a vital component in ECM of the dermis [58]. A preliminary study using cultured fibroblasts showed that topical application of HA prevented ROS injury attributed to radiation. In one study, HA significantly decreased the occurrence of serious skin injuries [59]. However, Pinnix et al. discovered that the discovered that the area administrated with oil was better than that administrated with HA [58].

### Biologic preparations

### Epidermal Growth Factor (EGF)

EGF is important in inducing the proliferation of fibroblasts, epidermal stem cells, and keratinocytes [60]. Reports have demonstrated that platelet, macrophages, and fibroblasts release EGF in acute wounds and adjuvant treatments [61]. EGF is capable of expediting the local healing of diabetic foot ulcers [62]. Kang et al. confirmed that the topical use of EGF down-regulated the incidence of grade 2 toxicities in patients with radiotherapy [63].

### Granulocyte Macrophage-Colony Stimulating Factor (GM-CSF)

GM-CSF is a lymphokine that facilitates the chemotacticity of monocytes into tissues, thus hampering the progress of macrophages. Macrophages secrete plasminogen activator in the presence of GM-CSF [64]. Patients administrated with steroids and GM-CSF had lower radiation dermatitis scores and less pain compared with those administrated with topical steroids alone [65].

### Pentoxifylline (PTX)

PTX refers to a competitive nonselective phosphodiesterase inhibitor. For its anti-inflammatory effect, it has been widely used in skin diseases; it can repair the radiation-induced damage by inhibiting the TGF-β expression [66]. Existing studies reported that a combination of pentoxifylline and alpha-tocopherol mitigates fibrosis for at least 6 months [67]. Microparticles loaded with pentoxifylline and succinate D-α-tocopherol act as a novel topical formulation that locally targets inflammatory cytokines and oxidation pathways, which are applied to the skin after local laser ablation [68].

### Plasma

Platelet-rich plasma (PRP) can improve the healing of skin wounds. Lee et al. studied the regeneration function of PRP by locally irradiating the back skin of mice. As revealed from the results, PRP enhances cell function via AKT signaling, thereby facilitating the regeneration of irradiated skin. The ability of PRP to promote skin healing is worth conducting clinical research and application [69].

### Interleukins (ILs)

Proinflammatory cytokines are critical to the adverse effects of early and late ionizing radiation. Inflammatory bodies are capable of maturing the pro-inflammatory cytokines (IL-6, IL-18, IL-22, and IL-1β), as well as exacerbating radiation damage [70–72]. It can be seen that inhibiting the expression of inflammatory factors can promote skin repair. However, IL-12 has a radioprotective effect on radiosensitive systems such as bone marrow and gastrointestinal tract and it is a potential mitigator of RIS [73].

### Superoxide dismutases (SODs)

SODs are endogenous enzymatic antioxidants that can act as an indicator to assay radiation-induced skin damage [74]. Existing studies suggested that oral administration of SOD-gliadin or SOD/catalase mimetic can prevent or mitigate radiation-induced skin fibrosis and injury in mice [75, 76].

### Pharmaceuticals

### Triethanolamine cream

Triethanolamine cream is a compound preparation with good hydration. Applying it to the damaged area of the patients' skin can drains and cleans the area, as well as effectively reducing patients’ skin dryness, decreasing body inflammation and edema response, facilitating patients’ body microcirculation and enhancing skin tolerance, thereby expediting the healing of the wound [77].

### Corticosteroids

Corticosteroids have anti-inflammatory effects. They are commonly employed to treat radiation-induced dermatitis because of its ability to prohibit radiation-induced cytokine proliferation [3]. Haruna et al. showed that the use of corticosteroids avoided the occurrence of wet desquamation and lowered the severity of RSI. The beneficial role of corticosteroids in preventing RSI has been verified [78]. Ho et al. demonstrated topical corticosteroids to be effective to reduce eczema peeling, reduce the frequency of serious skin toxicity and delay the occurrence of grade 3 dermatitis [79].

### Statins

On the whole, statins are adopted to treat hypercholesterolemia and prevent heart disease. They also have immunomodulatory, anti-inflammatory, metabolic, antioxidant and antibacterial characteristics [80]. Existing studies reported that statins can improve skin-related diseases and promote wound healing in ulcers [81]. Ghasemi et al. divided patients into the atorvastatin group and the placebo group for analysis, and the results showed that the reported use of atorvastatin mitigated primarily radiation-induced breast swelling, itching and pain [82].

### Trolamine

Trolamine is a topical oil-in-water emulsion widely used to treat RIS in the clinic. Trolamine acts as a nonsteroidal anti-inflammatory drug by recruiting macrophages and stimulating granulation tissue [83]. Multiple RCTs reported that triethanolamine in aloe vera–, vitamin-, and lipid-based creams or placebos could treat RD [84, 85]. As demonstrated by Abbas et al., the incidence of RTOG grade 3 dermatitis was down-regulated in patients with squamous cell carcinoma of the head and neck using triethanolamine emulsions [86]. Moreover, triethanolamine mitigated patient discomfort compared with β-sitosterol [56].

### Sucralfate

Sucralfate refers to the primary aluminum salt of sucrose octasulfate and an ordinary anti-ulcer drug when taken orally. Sucralfate exerts a significant barrier effect and exhibits anti-inflammatory and antibacterial properties; it can facilitate angiogenesis as well. Three studies were conducted to determine the clinical effect of sucralfate to treat RIS, with mixed results. In clinical studies, however, sucralfate significantly mitigated the severity of dermatitis or alleviated the symptoms of patients [87]. Fortunately, Kouloulias reported the conducive functions of sucralfate in a small and nonrandomized study [88].

### Metallic ointments and dressings

### Silver Sulfadiazine (SSD)

The topical antibacterial agent SSD is used primarily as a topical cream for serious burns. SSDs have displayed anti-inflammatory characteristics; they strengthen the barrier to protect the skin from infections [89]. When employed to manage RD, the overall RTOG dermatitis grade of SSD was lower than that of the control group [90].

### Silver nylon dressing

Silver nylon dressing is a nonadhesive nanocrystalline material. It is used clinically as a burn dressing. However, recent studies showed that nylon silver dressings helped control skin toxicity attributed to radiation [91, 92]. Compared with SSD, nylon silver dressing was superior in reducing the average dermatitis score and had a better effect. Compared with steroids, humectants, and SSD, it mitigated itching, pain and burning [91, 92].

### Silver-containing foam dressings with Safetac

Silver-containing foam dressings with Safetac is considered a transparent dressing that can gently be adhered to various skin surfaces. It can provide a moist healing environment for wounds and effectively protect and repair damaged skin [93], without interfering with the radiation dose. Several studies exploited this dressing to achieve good results [94, 95]. However, the sample size of the mentioned studies was generally small, the evidence strength was insufficient, and the credibility was low. For the mentioned reason, the conclusions were difficult to generalize directly to the clinic. Some researchers considered that self-adhesive soft silicone film dressings were not practical and suitable for all radiotherapy sites. Existing studies on self-adhesive soft silicone film dressing worldwide are basically limited to the prevention stage of radiation dermatitis [95, 96]. Some researchers exploited a novel type of soft silicone foam dressing to treat radiation-induced skin injuries and achieved effective results [97].

## Future directions

### Stem cells

Studies have reported that stem cells are a promising way to treat refractory skin damage. Human fetal skin stem cells (hFSSC) cover considerable stem and progenitor cells for development, which help treat skin damage. hFSSC is less antigenic and less likely to be rejected by transplant recipients [96]. Because of the mentioned characteristics, hFSSC can promote skin repair *in vivo* and is beneficial for skin damage [98]. Because of these characteristics, hFSSC can promote skin repair *in vivo* and is beneficial for skin damage [99]. Fetal skin in the uterus applies to scar-free tissue repair. Adult skin wounds heal slowly and form scars. The unique characteristics of hFSSC can promote scarless repair of wounds [100]. Stem cells can promote the repair of radiation-induced skin damage. Chao et al. used adipose-derived stem cells (ADSCs) as seed cells and HA as a carrier to prepare stem cell complexes to treat radiation-induced skin damage in rats [101]. Akita et al. performed a local injection of ADSCs covered with artificial skin to treat an elderly woman having developed chronic radiation–induced skin ulcers after radiotherapy of uterine cancer 40 years ago, achieving good results [102]. Recent studies identified that subcutaneous fat also exhibits endocrine functions; it can secrete various cytokines and participate in adjusting the biological behavior of epidermal cells and fibroblasts, thereby facilitating the wound healing quality [103]. ADSCs can promote the healing of radioactive skin injuries and provide hope for the treating radioactive skin injuries [104].

### Dermaprazole

Animal studies have shown that dermaprazole can improve the appearance of irradiated skin and accelerate wound healing. Histopathological results confirm that both prophylactic and therapeutic dermaprazole have anti-inflammatory and anti-fibrotic effects. Gene expression data indicate that dermaprazole downregulates some pro-oxidant, proinflammatory, and fibrotic genes. Desomeprazole's topical formulations can effectively mitigate skin inflammation and fibrosis [105].

### Subsequent research

Given the mentioned information, bevacizumab as a novel type of drug to treat radiation brain injury exhibits a higher overall treatment efficiency, and it may be more in-depth thinking and exploration in the future. For instance, some patients may show the recurrence of edema after being administrated with bevacizumab. Thus, a question is raised that whether it is effective to be applied bevacizumab again. If the answer is yes, how should we grasp its optimal dosage and course of treatment? The mentioned research can be deepened.

## Prognosis

Patients' prognosis will be generally determined by several factors, primarily based on the degree of radiation damage of grade 1, 2, 3, or 4 and the associated comorbidities [8].

## CONCLUSIONS

In brief, RSI is a more common radiation therapy complication. This type of skin protection and care is of great significance. Generally accepted guidelines for necrotic tissue management, infection prevention and treatment, wound exudate management, and re-assessment of treatment plans based on observation of wound progress should be conducted to treat full-thickness wounds resulting from delayed radiation injury. Patient education should consist of daily skin and wound care management and topical medications. More cost-effective protective measures exerting fewer side effects should be developed to effectively protect the interests of patients, ensure smooth chemotherapy, as well as improving the quality of life of patients.
